# The Effect of Adding Exogenous Bletilla Striata Polysaccharide on Kiwifruit Wine Quality

**DOI:** 10.3390/foods15142521

**Published:** 2026-07-16

**Authors:** Zhiqin Zheng, Chun Yi, Jun Zhao, Tian Zheng, Bin Guo, Tong Lin, Bing Xiong, Kangjie Yu, Yue Wang, Siyu Li, Xinying Zhang, Qiwen Li, Yi Ma

**Affiliations:** 1 School of Food and Liquor Engineering, Sichuan University of Science & Engineering, Yibin 644000, China; 2 Wuhu Green Food Industry Research Institute Co., Ltd., Wuhu 238300, China; 3 Anhui Province Key Laboratory of Conservation and Utilization for Dabie Mountain Special Bio-Resources, West Anhui University, Lu’an 237012, China; 4 Material Corrosion and Protection Key Laboratory of Sichuan Province, Sichuan University of Science & Engineering, Zigong 643000, China; 5 Yibin Wuliangye Xianlin Ecological Wines Co., Ltd., Yibin 644000, China; 6 Sichuan Provincial Alcoholic Beverage Research and Development Center, Chengdu 610095, China; 7 College of Agronomy, Northwest A&F University, Yangling 712100, China

**Keywords:** kiwifruit wine, *Bletilla striata* polysaccharide, antioxidant activity, aroma compounds, differentially expressed metabolites

## Abstract

Kiwifruit wine is valued for its nutrients and health benefits, but conventional brewing often results in a weak aroma due to precursor insufficiency and oxidative losses during fermentation. Therefore, adding natural polysaccharides with antioxidant and metabolic regulatory properties prior to fermentation may be a promising quality improvement strategy. *Bletilla striata* polysaccharide (BSP), a plant polysaccharide with multiple bioactivities, may have the potential to regulate kiwifruit wine quality. This study explored the regulatory effects of 100–400 mg/L BSP on the physicochemical properties, antioxidant activity, and aroma compounds of kiwifruit wine, which suggested its possible mechanism of action at the metabolite level. The results showed that the 300 mg/L BSP treatment achieved the best overall quality, maximizing the DPPH and ABTS radical scavenging rates and increasing the total relative abundance of volatile compound concentration by 28.69% compared with the control. Electronic nose testing, odor activity value analysis, and sensory evaluation all indicated that the 300 mg/L BSP group exhibited the best overall aroma profile, and non-targeted metabolomics further suggested that the 300 mg/L BSP addition was associated with the depletion of quinic acid, the accumulation of aromatic amino and succinic acids, and changes in lipid-related metabolites, which were correlated with enhanced aroma production. In conclusion, exogenous BSP addition appears to improve kiwifruit wine quality, and this study provides a theoretical basis for its application in fruit wine brewing.

## 1. Introduction

Kiwifruit wine is rich in nutrients and possesses a distinctive aroma [1]. It exhibits great potential in antioxidant and lipid metabolism regulation activities [2] and thus has broad market prospects. However, due to insufficient aroma precursors and oxidative losses during fermentation [3,4], traditional brewing techniques often produce kiwifruit wine with a thin mouthfeel and a weak aroma [5,6]. Therefore, improving kiwifruit wine quality has become a research focus.

In recent years, adding exogenous organic acids, natural polysaccharides, and polyphenols before fermentation to directionally regulate the fermentation metabolism process and wine quality has gradually become a research hotspot in the field of fruit wine brewing [7,8,9]. Natural polysaccharides, as safe and efficient biomacromolecules, can form complexes with the polyphenols and volatile organic compounds in fruit wine through non-covalent interactions, and these physicochemical interactions not only effectively inhibit polyphenol-mediated precipitation to reduce astringency but also target and regulate the release of core ester aromas, significantly improving the flavor and sensory harmony of fruit wine [10]. Natural polysaccharides can be classified as plant-based, animal-based, microbial, or seaweed-based according to their source [11]. To date, most studies on exogenous polysaccharides in fruit wine have focused on animal-derived (e.g., chitosan) and microbial-derived polysaccharides (e.g., yeast polysaccharides) [12,13]. In contrast, research on plant-derived polysaccharides in fruit wine fermentation remains limited, and their regulatory effects on wine quality have not been systematically explored. Thus, investigating plant-derived polysaccharides with suitable functional properties is a worthwhile endeavor for improving fruit wine quality.

*Bletilla striata* polysaccharide (BSP) is a water-soluble, naturally occurring plant polysaccharide extracted from the dried pseudobulb of the orchid *Bletilla striata*, and it belongs to the glucomannan family [14]. Studies have demonstrated that BSP is not a single chemical entity but rather a mixture of glucomannan components with varying molecular weights and structures [15,16]. It is primarily composed of mannose and glucose linked via 1,2- or 1,4-glycosidic bonds [14]. The antioxidant and antibacterial activities of BSP have been reported in previous studies [15,17]. For example, Zhu et al. [18] reported high free radical scavenging rates (87.6% for hydroxyl radicals, 83.9% for DPPH, and 71.5% for superoxide anions) at specific concentrations. BSP has also been shown to inhibit nutrient consumption in cherry tomatoes [19] and to suppress gray mold growth in grapes [20]. These findings suggest that BSP may possess properties relevant to fruit wine systems, but this has not been tested directly. However, current research on BSP has primarily focused on postharvest preservation and emulsion food systems [21,22], and its application in fruit wine fermentation has not yet been explored. Unlike yeast polysaccharides and chitosan, which have been extensively studied in fruit wine systems, BSP is a plant-derived glucomannan with a distinct monosaccharide composition and reported bioactivities. These structural and functional features make BSP a promising candidate for regulating kiwifruit wine quality, warranting systematic investigation.

To address the aforementioned research gaps, this study introduces, for the first time, BSP into a kiwifruit wine fermentation system. By supplementing the system with varying concentrations of BSP and employing techniques such as headspace solid-phase microextraction gas chromatography–mass spectrometry (HS-SPME-GC–MS) and ultra-high-performance liquid chromatography–quadrupole time-of-flight mass spectrometry (UHPLC-Q-TOF-MS), the regulatory effects of BSP on the physicochemical properties, antioxidant capacity, and aroma compounds of kiwifruit wine were systematically investigated. Furthermore, the metabolic regulatory mechanism of BSP in the kiwifruit wine fermentation system was elucidated at the metabolite level. Thus, this study provides a novel strategy for improving kiwifruit wine quality and offers theoretical and data-driven support for the innovative application of natural plant polysaccharides in the fruit wine brewing industry.

## 2. Materials and Methods

### 2.1. Materials and Reagents

Hongyang kiwifruits were purchased from Jiahong Industrial Park, Tianma Town, Chengdu, China. Sucrose (analytical grade) was obtained from a local commercial supplier. SY fruit wine yeast was purchased from Angel Yeast Co., Ltd. (Yichang, China). Rutin standard (purity ≥ 98.0%), gallic acid standard (purity ≥ 98.0%), 3,5-dinitrosalicylic acid (purity ≥ 98.0%), and potassium metabisulfite (purity ≥ 95.0%) were supplied by Shanghai Yuanye Biotechnology Co., Ltd. (Shanghai, China). Pectinase (analytical grade) was obtained from Henan Linbo Biotechnology Co., Ltd. (Zhengzhou, China). DPPH standard (purity ≥ 97.0%) was purchased from Hefei Bomei Biotechnology Co., Ltd. (Hefei, China). Folin–Denis reagent was obtained from Xiamen Haibiao Technology Co., Ltd. (Xiamen China). ABTS radical scavenging activity assay kit and ferric ion reducing antioxidant power (FRAP) assay kit were purchased from Beijing Box Biotechnology Co., Ltd. (Beijing, China) and Suzhou Gres Biotechnology Co., Ltd. (Suzhou, China), respectively. 2-Octanol (chromatographic grade), acetonitrile (HPLC grade), and methanol (HPLC grade) were supplied by Shanghai Maclean Biochemical Technology Co., Ltd. (Shanghai, China).

BSP (lot No. S27914-25g, purity ≥ 75%) was purchased from Shanghai Yuanye Biotechnology Co., Ltd. It is a water-soluble polysaccharide extracted from the tubers of *Bletilla striata*. The product was dissolved in water at 5 mg/mL. Insoluble impurities were removed by filtration. No further purification was performed. The manufacturer does not provide molecular weight or monosaccharide composition. In general, BSP is a glucomannan mainly composed of D-mannose and D-glucose, with molar ratios ranging from 1.5:1 to 4:1 depending on extraction methods, and molecular weight varying widely from approximately 28 kDa to over 400 kDa. Given the declared purity of ≥75%, the remaining fraction likely comprises co-extracted plant secondary metabolites, such as phenolic compounds and glycosides natively present in the *Bletilla striata* matrix. Therefore, the BSP used in this study represents a complex plant extract rather than a highly purified single polysaccharide.

### 2.2. Kiwifruit Wine Brewing

All experiments were conducted in a clean, sterile, and controlled standard laboratory environment, strictly adhering to food fermentation operation specifications and brewing process safety standards. These stable and reliable experimental conditions ensured the accuracy and reliability of the results. The fermentation method for kiwifruit wine was based on that described by Zheng et al. [23] with slight modifications. Fresh kiwifruits were selected, washed, peeled, and juiced. The juice was supplemented with 0.05% pectinase and hydrolyzed at 40 °C for 60 min. Sucrose was then added to adjust the sugar content to 20° Brix, and the pH was adjusted to 4.0. Potassium metabisulfite was added to achieve a final SO_2_ concentration of 60 mg/L. Subsequently, different concentrations of BSP were added, followed by inoculation with activated yeast (2% *v*/*v*). The juice was dispensed into 250 mL Erlenmeyer flasks and fermented at 22 °C. Each treatment was performed in triplicate. During fermentation, the weight of each flask was measured every 24 h. Fermentation was considered complete when the weight difference between two consecutive measurements was less than 0.3 g. The total fermentation period was 8 days. The experimental groups were as follows: control group (no BSP); group B1 (100 mg/L BSP); group B2 (200 mg/L BSP); group B3 (300 mg/L BSP); and group B4 (400 mg/L BSP).

To ensure a single-factor design with BSP concentration as the only variable, all kiwifruits were from the same harvest lot, and the homogenized juice was divided into fermentation vessels. Three independent fermentation batches were prepared for each treatment group (control, B1–B4) as biological replicates, with each batch fermented independently under identical conditions (22 °C, 8 days). For all subsequent physicochemical evaluations and instrumental analyses, each biological replicate was measured once, without additional technical replicates.

### 2.3. Basic Physicochemical Determination of Kiwifruit Wine

Soluble solids content was determined using a refractometer, pH was measured with a pH meter, and alcohol content was assessed using an alcohol meter. The contents of reducing sugars and total acidity were analyzed according to the Chinese national standard GB/T 15038-2006 [24].

### 2.4. Determination of Total Phenols

Following the method of Ma et al. [8] with slight modifications, a standard curve was first established. Six test tubes were each sequentially supplemented with 0.5 mL of distilled water and 0.5 mL of Folin–Ciocalteu reagent. After thorough mixing, 1 mL of gallic acid standard solution at concentrations of 0, 10, 20, 30, 40, and 50 µg/mL was added to each tube, respectively. Following a 3 min incubation, 1.5 mL of Na_2_CO_3_ solution with a volume fraction of 7.5% (*v*/*v*) was added. The mixture was then allowed to stand in the dark for 2 h, and the absorbance at 765 nm (OD_765_) was measured to derive the regression equation of the standard curve. The kiwifruit wine sample was diluted 20-fold. Following the same procedure, 1 mL of the diluted kiwifruit wine sample was used in place of the gallic acid standard solution. The total phenolic content was calculated using the standard curve. The resulting standard curve regression equation was: y = 40.414x − 0.0505, R^2^ = 0.9968, where y represents the absorbance at 765 nm and x represents the gallic acid concentration (µg/mL).

### 2.5. Determination of Total Flavonoids

With slight modifications to the method described by Hou et al. [25], a standard curve was first established. A rutin standard solution at a concentration of 0.2 mg/mL was prepared. Aliquots of 0, 2.0, 4.0, 6.0, 8.0, 10.0, and 12.0 mL of the rutin solution were transferred into seven 50 mL volumetric flasks, respectively. To each flask, 70% (*v*/*v*) anhydrous ethanol was added to a volume of 20 mL, followed by the addition of 2.0 mL of 4.5% (*w*/*v*) NaNO_2_ solution. After shaking and standing for 5 min, 2.0 mL of 10% (*w*/*v*) Al(NO_3_)_3_ solution was added, followed by another shaking and standing for 5 min. Finally, 4.0 mL of 1.0 mol/L NaOH solution was added, and the mixture was diluted to 50 mL with distilled water. The absorbance of each flask at 510 nm (OD_510_) was measured to obtain the regression equation of the standard curve. Subsequently, 1 mL of the kiwifruit wine sample was accurately pipetted into a 50 mL volumetric flask, and the remaining procedure was the same as that described for the standard curve. The total flavonoid content was calculated based on the standard curve. The resulting standard curve regression equation was: y = 0.0005x + 0.0129, R^2^ = 0.9974, where y represents the absorbance at 510 nm and x represents the rutin concentration (mg/mL).

### 2.6. Determination of Antioxidant Capacity

#### 2.6.1. DPPH Radical Scavenging Activity

The method of Ma et al. [8] was adopted with slight modifications. Briefly, 7.8864 mg of analytical-grade 2,2-diphenyl-1-picrylhydrazyl (DPPH) was dissolved in anhydrous ethanol and diluted to a final volume of 100 mL. The kiwifruit wine sample was diluted 40-fold, and 1.0 mL of the diluted sample was mixed with 3.0 mL of DPPH radical solution. The mixture was then incubated in the dark at 37 °C for 30 min. The absorbance of the reaction mixture was measured at 517 nm. Distilled water was used as a blank control. The DPPH radical scavenging activity of the sample was calculated using Formula (1):

(1) $$\mathrm{DPPH}\;\mathrm{free}\;\mathrm{radical}\;\mathrm{scavenging}\;\mathrm{activity}\left(\%\right)\;=\;\left(A_{0}\;-{\;A}_{1}\right)/A_{0}\;\times \;100$$

*A*_0_ represents the absorbance of the DPPH solution in water (blank), and *A*_1_ represents the absorbance of the sample in the DPPH solution.

#### 2.6.2. ABTS Radical Scavenging Activity

The scavenging capacity of 2,2′-azino-bis (3-ethylbenzothiazoline-6-sulfonic acid) (ABTS) was determined according to the instructions of the assay kit. The fruit wine sample was first diluted 10-fold, and the working solution and the application solution provided in the kit were prepared. An aliquot of 50 µL of the diluted sample, 100 µL of the application solution, and 850 µL of the working solution were thoroughly mixed and then incubated at room temperature in the dark for 6 min. The absorbance was measured at 405 nm. A blank control was prepared for each sample. The experimental results were calculated using Formula (2):

(2) $$\mathrm{ABTS}\;\mathrm{radical}\;\mathrm{scavenging}\;\mathrm{activity}\left(\%\right)\;=\;{(A}_{0}\;-\;{(A}_{2}\;-{\;A}_{1}))/A_{0}$$

*A*_0_ represents the absorbance of the blank tube, *A*_1_ represents the absorbance of the control tube, and *A*_2_ represents the absorbance of the sample tube.

#### 2.6.3. Ferric Reducing Ability of Plasma

FRAP was determined according to the kit instructions. The fruit wine sample was first diluted 10-fold. An aliquot of 680 µL of the chromogenic solution, 20 µL of the diluted sample, and 100 µL of distilled water were thoroughly mixed and incubated at room temperature for 10 min. The absorbance was measured at 590 nm. The blank was prepared by mixing 680 µL of the chromogenic solution with 120 µL of distilled water. The experimental results were calculated using Formula (3):

(3) $$\mathrm{Ferric}\;\mathrm{Reducing}\;\mathrm{ability}\;\mathrm{of}\;\mathrm{plasma}\left(µmol\;{\mathrm{FeSO}}_{4}/\mathrm{mL}\right)\;=\;1.95\;\times \;\left(\left(A_{1\;}-{\;A}_{0}\right)\;+\;0.0011\right)\;\times \;D$$

*A*_0_ represents the absorbance of the blank tube, *A*_1_ represents the absorbance of the sample tube, and *D* represents the dilution factor.

### 2.7. Determination of Volatile Compounds

HS-SPME-GC-MS analysis was performed using a Varian 3900 gas chromatograph coupled with a Varian Saturn 2100 ion trap mass spectrometer (Varian, Palo Alto, CA, USA). Volatile components in kiwifruit wine were semi-quantitatively determined by HS-SPME-GC-MS using a method slightly modified from Ma et al. [8]. HS-SPME extraction is affected by matrix components, compound polarity, volatility, and fiber adsorption. Thus, our reported concentrations are relative apparent values for semi-quantitative comparison.

An aliquot of 8 mL of kiwifruit wine was accurately transferred into a 15 mL headspace vial, followed by the addition of 2 g of NaCl and 20 µL of 2-octanol internal standard (8.19 mg/mL). The sample was preheated at 45 °C for 10 min, after which the conditioned microextraction fiber was inserted into the headspace vial, and the fiber was exposed (1.5 cm above the surface of the kiwifruit wine). Headspace adsorption was carried out for 35 min. After adsorption, the fiber was retracted and immediately transferred to the GC injection port for thermal desorption at 250 °C for 3 min.

GC conditions: DB-WAX capillary column (60 m × 0.25 mm × 0.25 µm); injection port temperature 250 °C; splitless injection mode. The temperature program was as follows: initial temperature 40 °C held for 5 min, then increased to 60 °C at 2 °C/min, then to 180 °C at 5 °C/min and held for 5 min, then to 230 °C at 10 °C/min and held for 10 min. Carrier gas: high purity helium at a constant flow rate of 1.2 mL/min.

MS conditions: electron impact ionization (EI); ion source temperature 230 °C; electron energy 70 eV; acquisition mode: full scan; MS quadrupole temperature 150 °C; solvent delay 3 min.

For qualitative analysis, we searched the mass spectra against the NIST/Wiley database. We retained hits with a similarity above 90%. To improve confidence, we calculated experimental retention indices (RI) using n-alkanes (C_8_–C_24_). We compared these RI values with literature data. We accepted compounds with a deviation (ΔRI) within 30 as tentative identifications. For semi-quantification, we estimated the relative concentration of each compound from its peak area ratio to the internal standard (2-octanol).

### 2.8. Odor Activity Value Calculation and Evaluation

To preliminarily screen for volatile compounds that may contribute to the aroma of kiwifruit wine, we calculated the odor activity value (OAV) for each compound. The formula is:

(4) $$\mathrm{OAV}\;=\;C_{i}/T_{i}$$

*C_i_* represents the quantitative concentration (µg/L) of the target volatile substance *i* in the sample, and *T_i_* represents the odor threshold (µg/L) of the compound in aqueous solution or in the corresponding ethanol–water matrix as reported in the literature.

When OAV > 1, the concentration of the compound exceeds its odor threshold, and it is considered a putatively odor-active compound that may contribute to the overall flavor of the fruit wine. The descriptions of the olfactory thresholds and aroma characteristics of each volatile compound in this study mainly refer to the threshold compilation by van Gemert [26], Fenaroli’s Handbook of Flavor Ingredients [27], the Flavornet database [28], and related literature [29].

It must be noted that since the concentrations were derived from semi-quantitative HS-SPME without standard calibration curves for each specific compound, the calculated OAVs represent theoretical estimates. Furthermore, odor thresholds are highly matrix-dependent. Therefore, OAVs in this study solely serve as a preliminary screening tool for potentially important compounds, and do not definitively prove their real sensory contribution without further validation via gas chromatography–olfactometry (GC-O).

### 2.9. Aroma Sensory Evaluation

Sensory evaluation was conducted on wine samples from each treatment group (control, B1–B4), focusing on orthonasal aroma attributes to complement and validate the instrumental analysis. This procedure was performed with reference to the method described by Wang et al. [30], with minor modifications. A total of 25 panelists (12 males and 13 females, aged 22–26) were recruited from the School of Food and Wine Engineering, Sichuan University of Science and Engineering. All panelists underwent an 8-week training program consisting of two 1.5 h sessions per week. The training covered fundamental theories of sensory evaluation, aroma identification of fruit wine, aroma description training, and aroma intensity ranking exercises, in accordance with GB/T 16291.1-2012 [31]. Two experienced sensory experts with national-level professional qualifications in fruit wine sensory evaluation participated throughout the training process and provided guidance.

After training, we conducted a series of screening tests. The tests followed the procedures in GB/T 16291.1-2012. They evaluated the panelists’ sensory skills. The screening included four tests: (1) odor identification and discrimination with standard references for olfactory sensitivity; (2) basic taste recognition for five basic tastes (sweet, sour, salty, bitter, and umami); (3) difference tests (triangle test and paired comparison test) for discrimination ability; and (4) descriptive tests for vocabulary and intensity scaling. Only panelists who passed all four tests were selected for the final panel. Based on these results, we chose six panelists (three males and three females). They joined the two experts to form an eight-member panel. The panel then discussed and selected seven aroma attributes for kiwifruit wine. These attributes were fruity, floral, acidic, alcoholic, sweet, herbal, and woody.

For each wine sample, a 10 mL aliquot was poured into a 30 mL standardized tasting cup, randomly coded with a three-digit number, and covered with a lid. The samples were presented to the panelists sequentially in a randomized order at room temperature (22 ± 1 °C), and each panelist conducted three replicate evaluations. A 5–10 min interval was maintained between evaluations of different samples to ensure olfactory sensitivity and assessment accuracy. Each panelist rated the intensity of each attribute on a 6-point scale ranging from 0 (not detectable) to 5 (extremely strong). All evaluations were performed in a controlled sensory panel room with constant temperature, adequate lighting, and odor-free conditions. All participants provided informed consent, and the sensory evaluation procedures were conducted in accordance with ethical guidelines. The qualification certificates of the two nationally certified experts are provided as Supplementary Materials.

### 2.10. Electronic Nose

E-nose (Shanghai Fenrui International Trade Co., Ltd., Shanghai, China) assessed the aroma profile’s similarity. Following the method of Diao et al. [32] with slight modifications, 20 mL of kiwifruit wine sample was placed in a sample vial for analysis. The data acquisition time was 240 s. After measurement, principal component analysis (PCA) and linear discriminant analysis (LDA) were performed on the data recorded by the electronic nose. The electronic nose sampling parameters are presented in Table S1.

### 2.11. Non-Targeted Metabolomics Analysis Method

Prior to the non-targeted metabolomics analysis, we evaluated the effects of different BSP concentrations (0, 100, 200, 300, and 400 mg/L) on the volatile profile of kiwifruit wine. This evaluation was based on HS-SPME-GC-MS analysis, electronic nose analysis, and sensory evaluation of aroma attributes. Among all treatments, the B3 group (300 mg/L) showed the highest levels of key volatile compounds, particularly esters and alcohols. It also exhibited the most distinct and intense aroma profile. Accordingly, 300 mg/L BSP was identified as the optimal concentration for enhancing kiwifruit wine aroma. To further investigate the metabolic basis underlying this improvement and to understand the metabolite changes associated with the best aroma performance, we selected the CK and B3 groups for non-targeted metabolomics analysis.

Non-targeted metabolomics analysis was performed using an Agilent 1290 Infinity LC system (Agilent Technologies, Santa Clara, CA, USA) coupled to an AB SCIEX Triple TOF 6600 mass spectrometer (AB SCIEX, Framingham, MA, USA). Non-targeted metabolomics analysis was performed on three biological replicates per group (control and B3, *n* = 3 per group).

Sample preparation: An appropriate amount of sample was added to a precooled methanol/acetonitrile/water solution (2:2:1, *v*/*v*/*v*). The mixture was vortexed, sonicated at low temperature for 30 min, and then allowed to stand at –20 °C for 10 min. After centrifugation at 14,000× *g* for 20 min at 4 °C, the supernatant was collected and dried under vacuum. For mass spectrometry analysis, the dried residue was redissolved in 100 µL of acetonitrile/water (1:1, *v*/*v*), vortexed, and centrifuged at 14,000× *g* for 15 min at 4 °C. The resulting supernatant was then injected for analysis.

Chromatographic conditions: Sample separation was performed on an Agilent 1290 Infinity ultra high performance liquid chromatography (UHPLC) system equipped with a C18 column. The column temperature was maintained at 40 °C, the flow rate was 0.4 mL/min, and the injection volume was 2 µL. The mobile phase consisted of solvent A (water containing 25 mM ammonium acetate and 0.5% formic acid) and solvent B (methanol). The gradient elution program was as follows: 0–0.5 min, 5% B; 0.5–10 min, B was linearly increased from 5% to 100%; 10.0–12.0 min, B was held at 100%; 12.0–12.1 min, B was linearly decreased from 100% to 5%; 12.1–16 min, B was maintained at 5%. Throughout the analysis, the autosampler was kept at 4 °C. To avoid the influence of instrument signal fluctuations, samples were analyzed continuously in a randomized order. Quality control (QC) samples were inserted into the sample sequence to monitor and evaluate system stability and experimental data reliability.

Mass spectrometry conditions: Primary and secondary mass spectra were acquired using an AB Triple TOF 6600 mass spectrometer. The electrospray ionization (ESI) source conditions after chromatographic separation were as follows: Ion Source Gas 1 (Gas1): 60; Ion Source Gas 2 (Gas2): 60; Curtain gas (CUR): 30; source temperature: 600 °C; IonSpray Voltage Floating (ISVF): ±5500 V (positive and negative modes). TOF MS scan range: *m*/*z* 60–1000 Da; product ion scan range: *m*/*z* 25–1000 Da; TOF MS scan accumulation time: 0.20 s/spectrum; product ion scan accumulation time: 0.05 s/spectrum. MS/MS data were acquired using information dependent acquisition (IDA) in high sensitivity mode, with declustering potential (DP): ± 60 V (positive and negative modes) and collision energy: 35 ± 15 eV. The IDA settings were as follows: exclude isotopes within 4 Da; candidate ions to monitor per cycle: 10.

The raw MS data were converted to mzXML format using ProteoWizard MSConvert (version 3.0.24164) before being imported into the freely available XCMS software (version 4.6.4). For peak picking, the following parameters were used: centWave with *m*/*z* = 10 ppm, peakwidth = c(10, 60), and prefilter = c(10, 100). For peak grouping, bw = 5, mzwid = 0.025, and minfrac = 0.5 were applied. CAMERA (Collection of Algorithms for MEtabolite pRofile Annotation) was used for the annotation of isotopes and adducts. From the extracted ion features, only variables with more than 50% of non-zero measurements in at least one group were retained. Metabolite identification was performed by comparing the accurate *m*/*z* values (error < 10 ppm) and MS/MS spectra with an in-house database established using available authentic standards.

### 2.12. Statistical Analysis

Data were analyzed using R software (version 4.5.2) and expressed as mean ± standard deviation (mean ± SD). Data normality was verified using the Shapiro–Wilk test, and homogeneity of variances was examined using Levene’s test. When one-way ANOVA indicated significant differences among groups, Duncan’s multiple range test was applied for post hoc pairwise comparisons. Results were denoted with superscript letters. For the two-group comparison (CK vs. B3) in the non-targeted metabolomics analysis, an independent samples Student’s *t*-test was applied, and statistical significance was set at *p* < 0.05. Other figures were generated using Origin 2026, R 4.5.2, and online platforms (https://www.metaboanalyst.ca/ and https://www.omicstudio.cn/tool, accessed on 15 April 2026).

## 3. Results

### 3.1. Analysis of Physicochemical Properties

The results of the physicochemical indicators are presented in Table S2, highlighting the varying effects of adding different BSP concentrations on the basic physicochemical properties of kiwifruit wine.

Regarding pH value, all BSP-supplemented groups had significantly higher pH values than the control group (*p* < 0.05), indicating that BSP addition was associated with increased pH. Soluble solids content was highest in the control group and lowest in the B2 and B4 groups after BSP treatment, suggesting that BSP addition was associated with changes in soluble solid content. Additionally, the control group had the lowest total acidity. Groups B2, B3, and B4 showed significantly higher total acidity than the control (*p* < 0.05), whereas group B1 showed no significant difference (*p* > 0.05), indicating that BSP addition at 200, 300, and 400 mg/L was associated with higher total acidity. No significant differences in alcohol content were observed among the groups (*p* > 0.05), indicating that the treatments did not interfere with the yeast’s alcoholic fermentation capacity. Regarding reducing sugars, only group B1 exhibited significantly higher levels than the control and the other treatment groups (*p* < 0.05), while the remaining groups showed no significant difference from the control (*p* > 0.05), suggesting that the 100 mg/L BSP treatment was associated with higher residual reducing sugar levels.

Overall, BSP addition optimized pH and total acidity levels without negatively affecting alcoholic fermentation. Specifically, groups B2 and B3 exhibited a relatively balanced effect in increasing acidity, reducing soluble solids, and maintaining residual sugars, making their treatment concentrations the most suitable for BSP addition.

### 3.2. Analysis of Total Phenolic and Flavonoid Contents

**Figure 1:**
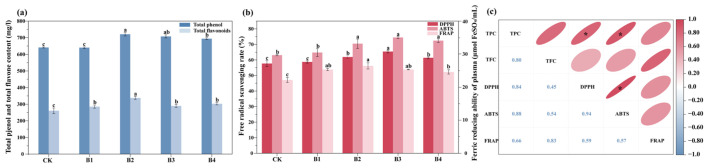
Effects of exogenous *Bletilla striata* polysaccharides on total phenolic content, total flavonoid content, and antioxidant capacity in kiwifruit wine. (**a**) Total phenolic and flavonoid contents in kiwifruit wine. Both contents are presented as bar charts and correspond to the left vertical axis (in mg/L). (**b**) Antioxidant capacity of kiwifruit wine. The DPPH and ABTS radical scavenging rates (%) are shown on the left vertical axis, while the FRAP value (µmol FeSO_4_/mL) is shown on the right vertical axis. (**c**) Pearson correlation analysis of total phenolic content, total flavonoid content, and antioxidant capacity indicators (DPPH, ABTS, and FRAP). Asterisks indicate significant correlations (* *p* < 0.05). Error bars represent the standard deviation (*n* = 3), and different lowercase letters indicate significant differences among the various BSP treatment groups (*p* < 0.05).

The results of total phenolic content (TPC) and total flavonoid content (TFC) determination are shown in Figure 1a. Adding exogenous BSP resulted in a modulatory effect on TPC and TFC in kiwifruit wine. Except for group B1, the TPC levels in the BSP-supplemented groups were significantly higher than that in the control group (*p* < 0.001). Additionally, the TFC levels in all experimental groups were also significantly higher than that in the control group (*p* < 0.001). With increasing BSP concentration, the TPC and TFC in kiwifruit wine generally increased initially and then decreased.

At a BSP addition concentration of 200 mg/L, both TPC and TFC reached their peak values of 720.89 ± 10.03 mg/L and 337.39 ± 8.45 mg/L, respectively. These values were significantly higher than those of the control group (*p* < 0.001). The influence of polysaccharide addition on phenolic content has also been documented in other fruit wines. For instance, Puerta García et al. [33] reported that adding polysaccharides to rosé wines increased flavanol and phenolic acid concentrations by 43.7% and 11.5%, respectively, relative to the control. Similarly, Curiel Fernández et al. [34] demonstrated that grape polysaccharides can affect the phenolic composition of wines. Although the present study did not directly investigate the underlying mechanism, one possible explanation is that BSP may help protect phenolic compounds from oxidative degradation during fermentation, as suggested by previous studies on polysaccharide–polyphenol interactions [10].

When the BSP concentration was increased to 300 mg/L and 400 mg/L, the TPC and TFC decreased. A similar concentration-dependent effect has been observed for other polysaccharides. A possible explanation is that excessive polysaccharide addition may induce polyphenol oligomer aggregation and precipitation [10,35,36] or encapsulation of phenolics within the polysaccharide matrix [37]. However, these possibilities have not been directly verified in the present wine system and warrant further investigation.

### 3.3. Analysis of Antioxidant Capacity

The results of antioxidant capacity determination are shown in Figure 1b. Adding exogenous BSP improved the antioxidant capacity of kiwifruit wine. As the BSP concentration increased, the antioxidant capacity generally increased first and then decreased, with different trends observed across the three assay systems.

The results of Pearson correlation analysis are presented in Figure 1c. FRAP showed no significant correlation with the radical scavenging indicators (DPPH and ABTS). In contrast, a strong positive correlation was observed between DPPH and ABTS (*r* = 0.94, *p* < 0.05). These results suggest that the antioxidant mechanisms underlying FRAP may differ from those of DPPH and ABTS, while DPPH and ABTS may share similar radical scavenging pathways. This finding is consistent with previous reports [38,39,40]. Accordingly, the subsequent discussion on the antioxidant capacity of kiwifruit wine in response to exogenous BSP addition is interpreted based on the distinct mechanisms of these three assays.

Group B2 exhibited the strongest FRAP, which was significantly higher than that of the control group (*p* < 0.05), and this trend was consistent with those of the TPC and TFC. The FRAP assay is based on the single-electron transfer (SET) mechanism, and its reducing capacity is positively correlated with the amount of electron donors such as polyphenols [38]. This trend is consistent with the fact that group B2 had the highest TPC, possibly because the FRAP results were largely driven by the phenolic composition.

In contrast, the maximum DPPH and ABTS radical scavenging rates were observed in group B3. Unlike FRAP, which primarily proceeds via SET, the scavenging of DPPH and ABTS radicals can also involve hydrogen atom transfer (HAT) [39,40]. BSP itself has been reported to possess antioxidant activity [15], and using density functional theory-based calculations, Hernandez Marin and Martinez [41] demonstrated that carbohydrates can scavenge free radicals through the HAT mechanism, with the hydrogen atom from hydroxyl groups serving as a direct donor. Thus, we speculate that the hydroxyl groups on the BSP molecular chain may act as hydrogen donors and contribute to its antioxidant activity. However, this mechanism is primarily supported by the literature and remains speculative in our study [41].

In group B3, the phenolic content decreased, possibly due to complexation with polysaccharides. Meanwhile, the remaining free BSP in the system may have contributed additional hydrogen-donating capacity. It is therefore possible that the HAT activity of BSP complemented that of the phenolic compounds, and their combined effect may account for the highest DPPH and ABTS scavenging rates observed in this group. However, this interpretation remains speculative and requires direct experimental verification.

When the BSP concentration reached 400 mg/L, one possible explanation is that excess polysaccharide molecules may have occupied most of the available active sites. This could have reduced their interaction with free radicals and potentially contributed to the concurrent decrease in DPPH, ABTS, and FRAP values.

### 3.4. Integrative Analysis of Aroma Profiles

#### 3.4.1. Analysis of Volatile Compounds

**Figure 2:**
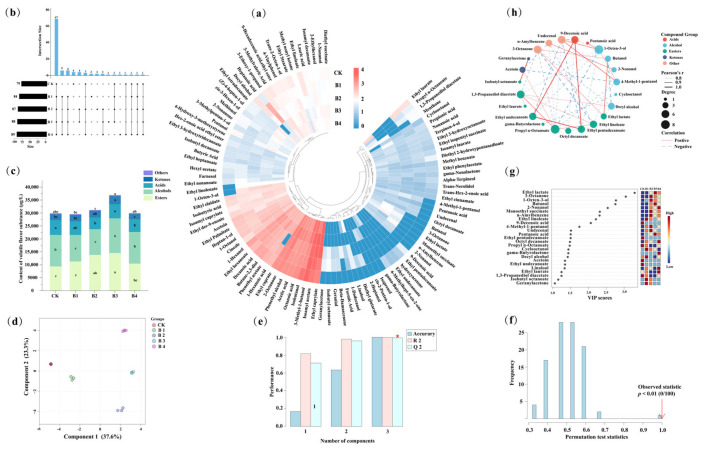
Effects of exogenous *Bletilla striata* polysaccharides on volatile substances in kiwifruit wine. (**a**) Heatmap of all volatile substances in kiwifruit wine; (**b**) Upset plot of all volatile substances in kiwifruit wine; (**c**) stacked bar chart of all volatile substances in kiwifruit wine; (**d**) principal component analysis (PCA) of all volatile substances in kiwifruit wine (PC1 vs. PC2, *p* < 0.05); (**e**) 5-fold cross-validation plot of the PLS-DA model; The red asterisk (*) indicates the optimal number of components selected based on the highest Q^2^ value; (**f**) 100-permutation test plot of the PLS-DA model; (**g**) variable importance in projection (VIP) plot of key volatile substances (VIP > 1); (**h**) correlation network of key volatile substances (|*r*| ≥ 0.7, *p* < 0.05). The sample size was *n* = 3, and different lowercase letters indicate significant differences among different *Bletilla striata* polysaccharide treatment groups (*p* < 0.05).

The volatile substances detected by HS-SPME-GC-MS were classified and summarized, and the macroscopic changes in the composition and content of volatile substances in each group are shown in Figure 2a. A total of 103 volatile substances were tentatively identified across all fruit wines, including 41 esters, 32 alcohols, 15 acids, 10 ketones, and 5 other volatile compounds. To further explore the effect of exogenous BSP addition on the composition and concentration of volatile substances in kiwifruit wine, stacked bar charts and Upset plots were generated, with the results presented in Figure 2b,c. Exogenous BSP addition exerted a regulatory effect on the volatile substances in kiwifruit wine, with both composition and concentration exhibiting different trends.

The Upset plot shows that 67 volatile substances, constituting the main flavor skeleton of kiwifruit wine, were common to all groups. The CK group contained 79 volatile substances. With increasing BSP concentration, the number of volatile substances showed an increasing trend, reaching a maximum of 89 in group B4. The stacked bar charts indicate that the volatile substances in the fruit wine were primarily composed of esters and alcohols. As the BSP concentration increased, the total estimated mass concentration of volatile substances first increased and then decreased, reaching a peak of 37,995.06 µg/L (expressed as 2-octanol equivalents) in group B3, which was 8470.88 µg/L higher than that of the control group. Furthermore, the total semi-quantitative levels of esters and alcohols in group B3 were significantly higher than those in the other groups (*p* < 0.05). This indicates that the addition of 300 mg/L BSP is associated with increased accumulation of volatile substances such as esters and alcohols. In contrast, when the BSP concentration reached 400 mg/L, the total volatile concentration decreased markedly. However, the variety of volatile compounds showed little change, with only one additional compound detected compared to group B3.

The observed differences between the 300 mg/L and 400 mg/L BSP treatments may reflect concentration-dependent effects on yeast metabolism. One possibility is that 300 mg/L BSP positively influences yeast metabolic activity, enhancing aroma accumulation [42]. In contrast, 400 mg/L BSP may alter the overall carbohydrate load in the fermentation broth and impose stress on yeast cells, analogous to high sugar conditions. Under such stress, yeast may suppress ester biosynthesis [43]. This could explain the sharp drop in volatile concentrations in group B4. However, this interpretation remains speculative and requires further verification in our system.

Having confirmed that BSP addition increases both the content and variety of volatile substances in kiwifruit wine, further multivariate statistical analysis was performed to verify the specificity among groups. Partial least squares discriminant analysis (PLS-DA) was conducted on the data, and the results are presented in Figure 2d, with the different treatment groups effectively separated in the score plot. Components 1 (40.8%) and 2 (28.8%) jointly explained a substantial portion of the variance, indicating good discriminatory power. To ensure the reliability of the model and guard against overfitting, a 5-fold cross-validation and a 100-permutation test were performed. As shown in Figure 2e, the model exhibited excellent explanatory and predictive capabilities, characterized by high *R^2^* and *Q^2^* values. Furthermore, the 100-permutation test (Figure 2f) yielded a highly significant result (*p* < 0.01), confirming the statistical robustness of the PLS-DA model. The CK group was independently located in the lower left corner and was clearly separated from all other groups along the first principal component. This further demonstrates that BSP addition not only alters the concentration of volatile substances but also reshapes their overall compositional profile.

To identify the key volatile substances responsible for the differences among groups, the variable importance in projection (VIP) of volatile substances was introduced. A VIP threshold greater than one was applied to select volatile substances that contributed significantly to the classification of the PLS-DA model, and the results are presented in Figure 2g. A total of 26 key volatile substances exhibited VIP values exceeding one, among which ethyl lactate, 3-octanone, 1-octen-3-ol, butanol, 2-nonanol, monoethyl succinate, n-amylbenzene, ethyl linoleate and 9-decenoic acid, made relatively large contributions. The relative abundance of color blocks on the right indicates that esters underwent pronounced changes following BSP treatment. Additionally, long-chain fatty acid ethyl esters, such as ethyl pentadecanoate and ethyl undecanoate, showed lower relative abundances in the BSP-treated groups, while other esters, including ethyl lactate and monoethyl succinate, accumulated to higher levels. Alcohols such as 1-octen-3-ol and linalool showed an increasing trend in groups B2, B3, and B4, while acids such as palmitic acid generally decreased. These dramatic fluctuations in key volatile substances are crucial for distinguishing differences among the groups.

To further explore the intrinsic relationships among key volatile substances identified by VIP analysis, we constructed a correlation network based on Pearson correlation coefficients (|*r*| ≥ 0.7, *p* < 0.05). The results are shown in Figure 2h. According to node degree values, propyl n-octanoate, ethyl undecanoate, ethyl pentadecanoate, 1,3-propanediol diacetate, 9-decenoic acid, 1-octen-3-ol, 4-methyl-1-pentanol, and 3-octanone exhibited larger node degrees. These compounds acted as core hubs, connecting numerous key volatile substances. This indicates that under BSP treatment, fatty acid metabolism, esterification, and related alcohol/ketone conversion pathways play a central role in regulating the overall volatile profile.

Strong positive correlations were observed among specific long-chain esters and acids. For example, propyl n-octanoate showed highly significant positive correlations with ethyl pentadecanoate and 9-decenoic acid (*p* < 0.05). Similarly, 1,3-propanediol diacetate was positively correlated with ethyl linoleate and ethyl lactate (*p* < 0.05). This suggests a coordinated accumulation pattern, likely driven by shared precursors in lipid degradation and esterification pathways.

Interestingly, no direct significant correlation was found between 1-octen-3-ol and 3-octanone. However, both compounds showed significant negative correlations with core ester and acid nodes, including propyl n-octanoate, ethyl undecanoate, and 9-decenoic acid (*p* < 0.05). Previous studies indicate that these eight-carbon volatiles are metabolically linked through the lipoxygenase hydroperoxide lyase (LOX-HPL) pathway during lipid degradation [44,45,46]. Their synchronized negative correlations with major esters suggest that exogenous BSP may alter the overall intracellular carbon flux. In the complex metabolic network of *Saccharomyces cerevisiae*, different product pathways compete for common precursors (e.g., acetyl-CoA) and cofactors (e.g., NADH) [47,48,49]. BSP addition likely shifts this equilibrium, competitively promoting the biosynthesis of specific fatty acid derivatives and complex esters while suppressing the accumulation of these higher alcohols and ketones. However, the exact mechanisms by which BSP influences these metabolic changes require further transcriptomic or metabolomic investigation.

#### 3.4.2. Odor Activity Value Analysis

Because different volatile substances have different sensory thresholds, their contribution to fruit wine aroma cannot be directly inferred from concentration levels alone. Therefore, we used the odor activity value (OAV) as a tool for preliminary screening of potentially odor-active compounds. The OAV results are presented in Supplementary Table S3, and a total of 31 volatile substances with an OAV ≥ 1 in at least one sample group were screened as putative aroma-active substances that may contribute to the aroma of kiwifruit wine, including 12 esters, 11 alcohols, 6 ketones, 1 acid, and 1 phenol.

Regarding esters, the OAV of isoamyl acetate increased from 163.63 ± 29.47 in the CK group to 295.78 ± 35.29 in the B3 group; ethyl hexanoate increased from 27.69 ± 4.1 to 61.33 ± 10.56; and ethyl decanoate increased from 93.43 ± 9.88 to 129.65 ± 16.26. However, in the B4 group, the OAVs of the above esters all decreased significantly (*p* < 0.05), exhibiting a trend of first increasing and then decreasing. Ethyl octanoate showed high OAVs (ranging from 285.16 to 292.29) in groups B2–B4, which were significantly higher than that in the CK group (*p* < 0.05).

Regarding alcohols, the OAVs of both hexanol and leaf alcohol (cis-3-hexen-1-ol) peaked in group B3, at 69.87 ± 9.82 and 5.94 ± 0.14, respectively, which are typically associated with grassy and fresh notes. In contrast, 1-octen-3-ol was detected only in BSP-supplemented groups (OAV 8.54–15.19). This result suggests that BSP addition may be related to its presence. However, further investigation is needed to determine whether this compound is newly induced or was simply below the detection limit in the CK group.

Regarding ketones, the OAV of 2-octanone increased from 101.06 ± 12.44 in the CK group to 156.91 ± 16.81 in the B3 group. However, 3-octanone was not detected in the CK or B1 group but was detected in groups B2–B4, further suggesting that BSP addition may have contributed to the formation of these compounds.

#### 3.4.3. Sensory Evaluation

**Figure 3:**
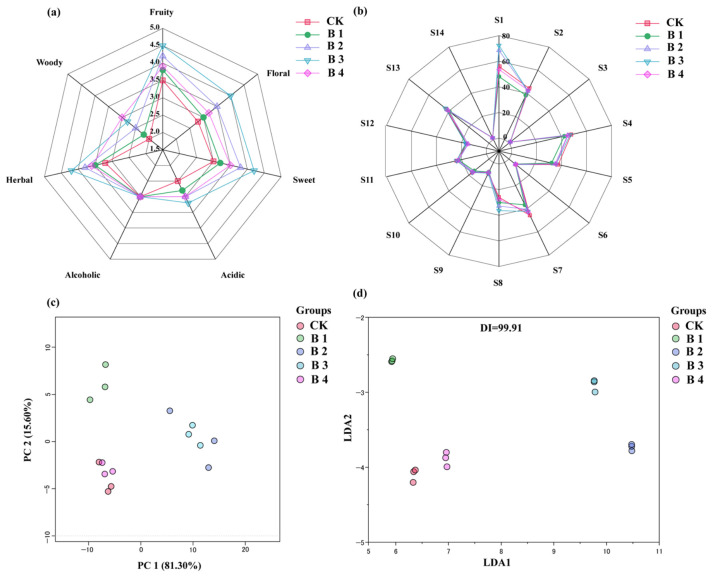
Effects of exogenous *Bletilla striata* polysaccharides on aroma profiles of kiwifruit wine evaluated via sensory analysis and an electronic nose. (**a**) Spider web diagram of sensory evaluation scores for seven aroma attributes; (**b**) radar chart of electronic nose sensor responses; (**c**) principal component analysis (PCA) of electronic nose data; (**d**) linear discriminant analysis (LDA) of electronic nose data. The sample size was *n* = 3.

Sensory evaluation was conducted to assess the orthonasal aroma attributes of kiwifruit wines with different BSP addition levels, and the results are presented as a radar chart in Figure 3a. For all seven aroma attributes, the intensity scores generally increased with BSP addition up to 300 mg/L and then slightly decreased at 400 mg/L. Among all groups, the B3 group showed the highest scores for fruity (4.5), floral (4.0), sweet (4.2), and herbal (4.2) attributes, while the alcoholic aroma score remained relatively stable across all groups (approximately 3.0), consistent with the measured alcohol content. In contrast, the woody aroma score gradually increased with rising BSP concentration and reached its highest value in the B4 group (3.0). Overall, the B3 group exhibited the highest aroma intensity among all treatments. These sensory evaluation results were in good agreement with the HS-SPME-GC-MS, OAV, and electronic nose analyses, thus corroborating the instrumental findings.

#### 3.4.4. Electronic Nose Analysis

The volatile aroma profiles of kiwifruit wines with different BSP addition levels were analyzed using an electronic nose, and the results are shown in Figure 3b. The radar chart patterns of the wines in each group were similar, indicating that the basic flavor skeleton of the wines did not change significantly. Compared with the control (CK) group, the radar chart profile area of group B1 was significantly reduced. Specifically, the mean response value of the S1 (ammonia) sensor decreased from 56.00 to 48.14, while that of the S8 (volatile organic compounds) sensor increased from 26.05 to 30.07. As the BSP addition level further increased, the response values of all sensors gradually rose. In group B3, the mean response value of the S1 sensor reached a peak of 72.36, representing a 29.2% increase compared with the CK, and the mean response value of the S8 sensor further increased to 32.43, a 24.5% increase relative to the CK. At this concentration, the radar chart profile area was the largest and most complete. These results suggest that the 100 mg/L BSP addition may have been associated with decreased responses to nitrogen-containing volatiles and increased responses to volatile organic compounds. When the BSP concentration was increased to 300 mg/L, the response values of both the S1 and S8 sensors were enhanced, and the overall radar chart profile area reached its maximum, which is consistent with the finding from volatile substance analysis that the aroma compounds in group B3 exhibited the best OAV performance.

PCA and LDA were used to analyze the sensor response data, and the PCA results are shown in Figure 3c. The cumulative variance contribution rate of the first two principal components (PC1 and PC2) reached 96.90% (PC1 = 81.30%, PC2 = 15.60%), indicating that they adequately captured most of the information in the original data. The sample points of groups CK and B1 were located close to each other, both distributed on the negative half-axis of PC1, suggesting that the addition of 100 mg/L BSP can affect the overall aroma profile of kiwifruit wine. This corresponds to the observation in the radar chart analysis, which showed that the profile area of group B1 was smaller than that of the CK. Groups B2 and B3 were located close to each other on the positive half-axis of PC1, indicating similar aroma profiles, which is consistent with the volatile substance analysis results.

The LDA results are presented in Figure 3d, with a DI value of 99.91, indicating that the LDA model has good discriminative ability for kiwifruit wines treated with different BSP concentrations. Compared with PCA, the LDA plot showed a clearer separation between treatment groups in the two-dimensional space. The control (CK) group was completely separated from the other groups, with group B1 independently distributed in the upper left region, while groups B2 and B3 were clustered on the right. These results indicate that BSP treatments at 200 and 300 mg/L achieved maximum separation from the CK group. Additionally, the 300 mg/L treatment maintained the distinguishing effect while exhibiting more compact clustering and a more stable aroma profile. This corroborates the conclusion from the volatile substance analysis that group B3 showed the best OAV performance for aroma compounds.

The complementary analytical approaches used in this study consistently showed that the B3 treatment exhibited the most desirable aroma profile. In terms of instrumental analysis, HS-SPME-GC-MS revealed that the B3 group had the highest relative abundance of volatile compounds, especially esters and alcohols. OAV calculation further confirmed that this group contained the most compounds with OAV > 1. Electronic nose analysis showed that the B3 group exhibited the strongest sensor responses in the principal component analysis (PCA) plot and had the clearest separation from the other treatment groups. Sensory evaluation of aroma also corroborated these results, with the B3 group scoring highest in fruity, floral, sweet, and herbal attributes. Overall, the combined results of instrumental semi-quantitative analysis, OAV screening, electronic nose profiling, and sensory aroma assessment support the conclusion that 300 mg/L BSP is the optimal concentration for enhancing the overall aroma of kiwifruit wine.

### 3.5. Non-Targeted Metabolomics Analysis

Based on HS-SPME-GC-MS analysis, electronic nose analysis, and sensory evaluation of aroma attributes, the B3 treatment group outperformed the other groups. It showed greater variety and higher concentrations of key volatile compounds, such as esters and alcohols. It also exhibited the strongest aroma intensity. Therefore, 300 mg/L BSP was identified as the optimal addition concentration for enhancing kiwifruit wine aroma. Given that this concentration exhibited the most pronounced regulatory effect on volatile flavor compounds, we further employed UHPLC-Q-TOF-MS technology to conduct a non-targeted metabolomics analysis of the CK and B3 groups. The aim was to explore the impact of the 300 mg/L BSP intervention on non-volatile metabolites in the kiwifruit wine fermentation system and to elucidate the underlying mechanism by which BSP influences it at the metabolite level.

**Figure 4:**
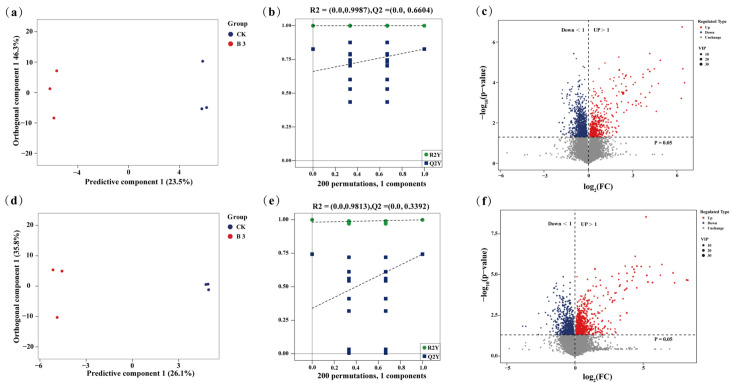
Integrated metabolomic workflow for screening differential metabolites in kiwifruit wine following exogenous *Bletilla striata* polysaccharide intervention. The analytical pipeline is sequentially structured to ensure rigorous data mining: (**a**,**d**) OPLS-DA score plots (positive and negative ion modes, respectively) employed for supervised pattern recognition to visualize global metabolic separation between treatment groups; (**b**,**e**) corresponding permutation tests (200 iterations) performed to validate the reliability and predictive power of the OPLS-DA models, thereby preventing overfitting; The dashed lines represent the linear regression lines of the R^2^Y and Q^2^Y values obtained from the permutation tests; and (**c**,**f**) volcano plots integrating univariate (*p* < 0.05) and multivariate (VIP > 1) statistical thresholds to pinpoint significantly altered metabolites as potential biomarkers. All data were derived from *n* = 3 biological replicates per group.

Orthogonal partial least squares discriminant analysis (OPLS-DA) was performed based on the non-targeted metabolomics data acquired via UHPLC-Q-TOF-MS. The OPLS-DA model was validated through 200 permutation tests, as shown in Figure 4b,e, which ruled out the risk of overfitting and ensured the reliability of subsequent analyses. As shown in Figure 4a,d, regardless of the ion mode (positive ion, POS; or negative ion, NEG), the BSP-supplemented group and the control group exhibited clear spatial separation along the principal component axis, indicating that BSP addition was associated with distinct metabolic profiles in the kiwifruit wine.

The volcano plot results are presented in Figure 4c,f. Using the screening criteria of (*p* < 0.05) and VIP > 1, over 40,000 metabolic features were detected in both POS and NEG modes, among which 346 and 697 features were identified as significantly different metabolites, respectively. However, among these numerous significantly different metabolites, only 51 could be structurally identified (as listed in Supplementary Table S4). Therefore, subsequent analyses focused on these 51 significantly differentially expressed metabolites.

**Figure 5:**
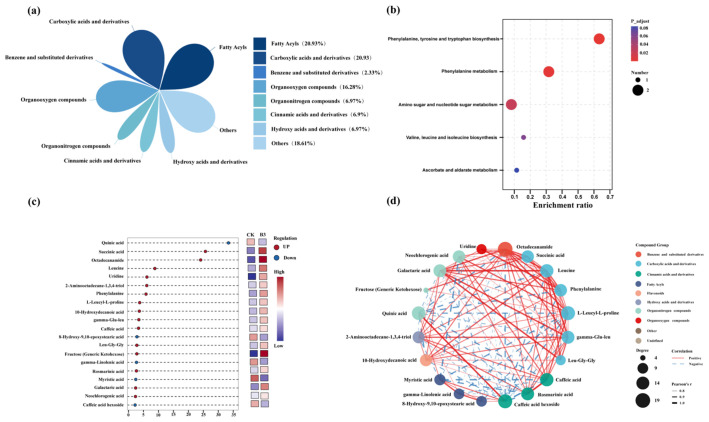
Analysis of the 43 significantly differential metabolites identified in kiwifruit wine. (**a**) Classification of these metabolites at the class level; (**b**) KEGG pathway enrichment bubble plot of these metabolites; (**c**) bar plot of the top 20 VIP values; (**d**) correlation network of the top 20 significantly differential metabolites based on the VIP ranking (|*r*| ≥ 0.8, *p* < 0.05). The sample size was *n* = 3.

The identification results from the two ionization modes were merged and deduplicated, and the 51 significantly differentially expressed metabolites were categorized at the class level according to the chemical classification system of the Human Metabolome Database, as shown in Figure 5a. These metabolites were mainly categorized as fatty acyls (20.93%), carboxylic acids and derivatives (20.93%), and organooxygen compounds (16.28%).

KEGG pathway enrichment analysis was performed on the 43 significantly differentially expressed metabolites, and the results are presented in Figure 5b. These metabolites were significantly enriched in the biosynthesis of phenylalanine, tyrosine, and tryptophan, as well as in the phenylalanine metabolic pathway (*p* < 0.05). In the present study, the enrichment of these pathways suggests that BSP treatment was associated with changes in the metabolic pathways related to aroma precursor production. Previous studies have shown that aromatic amino acids are important precursors for the synthesis of higher alcohols and characteristic esters by *Saccharomyces cerevisiae* via the Ehrlich pathway in alcoholic fermentation systems [50,51].

Specific amino acids directly involved in aroma synthesis, including phenylalanine and leucine, were significantly up-regulated following BSP addition (Figure 5c). The metabolism of these amino acids is closely linked to ester biosynthesis during alcoholic fermentation. Through the Ehrlich pathway, *Saccharomyces cerevisiae* converts branched-chain amino acids (e.g., leucine) and aromatic amino acids (e.g., phenylalanine) into higher alcohols via sequential transamination, decarboxylation, and reduction reactions [50]. Specifically, leucine catabolism yields isoamyl alcohol, while phenylalanine is converted to 2-phenylethanol. These higher alcohols serve as key precursors for ester formation. They are subsequently acetylated by alcohol acetyltransferases using acetyl-CoA to produce acetate esters, such as isoamyl acetate (fruity aroma) and 2-phenylethyl acetate (floral aroma) [52]. Therefore, the elevated levels of phenylalanine and leucine observed in this study suggest that BSP treatment may increase the intracellular pool of these precursors, which could in turn promote the biosynthesis of volatile esters and enhance the overall aroma profile. However, these interpretations are based on correlation analysis and remain speculative. Further studies are needed to confirm the underlying mechanisms.

Furthermore, the enrichment of the amino sugar and nucleotide sugar metabolic pathways is noteworthy, as nucleotide sugars are core donors for the synthesis of polysaccharides that constitute the yeast cell wall [53]. This suggests a potential link between BSP addition and changes in yeast cell wall-related metabolism. Studies have shown that glucomannan can cross-link with pectin via hydrogen bonds, forming a dense chain entanglement network and significantly increasing system viscosity [54]. Wang et al. [55] further confirmed that BSP exhibits shear-thinning properties in solution and produces a synergistic thickening effect when mixed with other polymers, and such increases in microviscosity are directly related to the fullness and richness perceived in wine sensory evaluations [56].

To further elucidate the interaction mechanisms among the significantly differentially expressed metabolites, the top 20 metabolites ranked by VIP value were first selected as core differentially expressed metabolites. Pearson correlation analysis was then performed on these metabolites using thresholds of |*r*| ≥ 0.8 and (*p* < 0.05). Based on the results, a correlation network was constructed (see Figure 5c,d). Based on both the VIP values of each metabolite and their positions in the correlation network, five significantly differentially expressed metabolites with high VIP values and central network positions were ultimately selected: quinic acid (VIP = 33.42, node degree = 13), succinic acid (VIP = 25.70, node degree = 13), leucine (VIP = 8.79, node degree = 15), phenylalanine (VIP = 5.84, node degree = 13), and octadecanamide (VIP = 24.12, node degree = 19). Subsequent analyses focused on these five key differentially expressed metabolites.

The network diagram shows that under BSP intervention, free quinic acid in the matrix was largely consumed, exhibiting a strong negative correlation with significantly upregulated amino acids, notably phenylalanine and leucine (*p* < 0.05). Quinic acid is a characteristic organic acid of kiwifruit [57]. In *Saccharomyces cerevisiae*, quinic acid metabolism is closely linked to the shikimic acid pathway, which provides carbon skeletons for aromatic amino acid biosynthesis [58]. The marked consumption of quinic acid coupled with phenylalanine accumulation suggests that BSP treatment may enhance metabolic flux through the shikimic acid pathway, potentially supporting the synthesis of aromatic precursors. Concurrently, the upregulation of leucine—a branched-chain amino acid derived from pyruvate metabolism—may hint at a broader impact on yeast nitrogen metabolism under BSP treatment. Crucially, these metabolic shifts are likely associated with aroma formation. Under anaerobic conditions, yeast catabolizes phenylalanine and leucine via the Ehrlich pathway through sequential transamination, decarboxylation, and reduction, yielding 2-phenylethanol (floral notes) and isoamyl alcohol (fruity notes), respectively [50]. These higher alcohols could serve as substrates for alcohol acetyltransferases, potentially leading to increased biosynthesis of volatile acetate esters. Thus, the accumulation of these specific amino acids offers a plausible mechanistic explanation for the enhanced aroma profile observed in the BSP-treated wine, though direct causal links remain to be verified.

Furthermore, succinic acid, as a highly interconnected core node, was upregulated. In the anaerobic environment of fruit wine brewing, the complete tricarboxylic acid (TCA) cycle in *Saccharomyces cerevisiae* is disrupted, and succinic acid is primarily synthesized via the reductive branch of the TCA cycle, i.e., the reduction of oxaloacetate to succinate through malate and fumarate [59]. This reductive pathway is considered a critical metabolic sink for regenerating NAD^+^, which helps maintain intracellular redox balance during glycolysis [59]. The upregulation of succinic acid suggests that BSP intervention may alter the redox status or glycolytic flux of the yeast, potentially increasing the demand for NAD^+^ regeneration. Biologically, this metabolic adjustment could help ensure yeast viability under fermentation stress. Sensorially, the accumulation of succinic acid has been associated with acidic, salty, and slightly bitter flavor notes in fermented beverages [60], which may contribute to taste complexity.

The network diagram revealed a strong negative correlation between lipid-related and phenolic acid-related metabolites in the fermentation system. In terms of taxonomy, cinnamic acid and its derivatives (phenolic acids), represented by rosmarinic acid and caffeic acid, exhibited strong positive correlations among themselves. Additionally, fatty acyls, represented by myristic acid and γ-linolenic acid, also showed a high degree of positive internal correlation. However, these two groups displayed a significant negative correlation with each other (*p* < 0.05). In the microenvironment of fruit wine fermentation, polyunsaturated fatty acids are essential components for yeast to maintain cell membrane fluidity and viability [61], and the negative correlation between phenolic acids and fatty acids observed in this study may reflect the metabolic interplay between lipid metabolism and secondary phenolic metabolism under BSP intervention.

Octadecanamide, as the node with the highest degree in the network, showed a strong positive correlation with ten metabolites, including succinic acid. The coordinated changes between octadecanamide and succinic acid suggest that these metabolites may have potential value as candidate biomarkers for quality changes during fermentation.

In summary, the correlation network analysis suggested that BSP intervention was associated with metabolic alterations in the kiwifruit wine fermentation system. A plausible explanation is that the depletion of quinic acid, coupled with the concurrent accumulation of phenylalanine and leucine, provides a reasonable upstream biochemical basis for the enhanced downstream biosynthesis of higher alcohols and esters observed in the B3 group. Meanwhile, the accumulation of succinic acid may have influenced the redox status and sensory complexity of the wine. Although these metabolic changes were highly consistent with the improvements in antioxidant capacity and volatile ester profiles, the precise enzymatic regulatory mechanisms driven by BSP remain to be further validated.

## 4. Discussion

This study investigated the effects of pre-fermentation BSP addition (100–400 mg/L) on kiwifruit wine quality. The results showed that the optimal supplementation level was 300 mg/L. At this concentration, the normal fermentation process remained unaffected. BSP significantly enhanced the overall antioxidant activity of the wine and yielded the most favorable aroma profile. Sensory evaluation confirmed that the B3 group attained the highest intensity scores for fruity, floral, sweet, and herbal notes. Non-targeted metabolomics analysis revealed that BSP addition was associated with changes in the metabolic landscape, including quinic acid depletion, accumulation of aromatic amino acids, and alterations in organic acid and lipid metabolism. In conclusion, pre-fermentation BSP addition at 300 mg/L is an effective strategy for improving the antioxidant capacity and aroma quality of kiwifruit wine.

From a practical perspective, the application of 300 mg/L BSP presents a promising technical strategy for the commercial production of high-quality, functional fruit wines. However, its industrial implementation may face certain limitations. Economically, the current costs associated with the extraction and purification of BSP could increase overall production expenses. Technically, achieving uniform dispersion and dissolution of this viscous polysaccharide in large-scale commercial fermentation tanks may present engineering challenges. More importantly from a safety standpoint, the commercial BSP preparation used herein is a complex matrix (≥75% purity) containing uncharacterized non-polysaccharide components. Although *Bletilla striata* has a long history of traditional use, rigorous toxicological and nutritional profiling of these co-extracted metabolites is strictly required before such extracts can be safely applied as additives in commercial food systems.

Finally, several limitations of the current study must be acknowledged. First, the experiments were conducted strictly at a laboratory scale using a single kiwifruit variety, which may not fully capture the complexities of industrial-scale fermentation or varietal differences. Second, the sensory evaluation relied on a trained panel consisting of only eight members, and broader consumer acceptance was not investigated. Furthermore, the long-term storage stability of the enhanced aroma and antioxidant profiles remains unevaluated. Future work should address these limitations through pilot-scale trials, comprehensive shelf-life evaluations, and large-scale consumer testing. Additionally, comparative studies with other polysaccharides (e.g., yeast mannoproteins and β-glucan) and the inclusion of physicochemical controls (such as viscosity adjustment) will be valuable to better elucidate the specific mechanisms and practical advantages of BSP in enology.

## Data Availability

The original contributions presented in this study are included in the article/Supplementary Materials. Further inquiries can be directed to the corresponding author.

## Supplementary Materials

The following supporting information can be downloaded at: https://www.mdpi.com/article/10.3390/foods15142521/s1, Table S1: Sensors and their corresponding performance descriptions; Table S2: Basic physicochemical properties of kiwifruit wine; Table S3: Odor activity values of key aroma-active compounds identified in kiwifruit wine; Table S4: Relative abundance of significantly differential metabolites.

## Abbreviations

The following abbreviations are used in this manuscript:

BSP	*Bletilla Striata* Polysaccharide
DPPH	2,2-Diphenyl-1-picrylhydrazyl
ABTS	2,2′-Azino-bis (3-ethylbenzothiazoline-6-sulfonic acid)
FRAP	Ferric Reducing Antioxidant Plasma
HS-SPME-GC–MS	Headspace Solid-phase Microextraction Gas Chromatography–mass
UHPLC-Q-TOF-MS	Ultra-high Performance Liquid Chromatography–quadrupole Time-of-flight Mass
E-nose	Electronic Nose
PCA	Principal Component Analysis
LDA	Linear Discriminant Analysis
TPC	Total Phenolic Content
TFC	Total Flavonoid Content
HAT	Hydrogen Atom Transfer
OAV	Odor Activity Valu
LOX-HPL	Lipoxygenase Hydroperoxide Lyase
VIP	Variable Importance in Projection
PLS-DA	Partial Least Squares Discriminant Analysis
OPLS-DA	Orthogonal Partial Least Squares Discriminant Analysis
NADH	Nicotinamide Adenine Dinucleotide
CoA	Coenzyme A
POS	Positive Ion
NEG	Negative Ion
TCA	Tricarboxylic Acid Cycle
