# Supplementary material for: The Effect of Adding Exogenous Bletilla Striata Polysaccharide on Kiwifruit Wine Quality
Source: Foods. 2026 Jul 16;15(14):2521. doi: 10.3390/foods15142521 (PMC13409640; doi:10.3390/foods15142521)
Supplement: Supplementary file 1 [file foods-15-02521-s001.zip › foods-4395159-supplementary.pdf]

Table S1. Sensors and their corresponding performance descriptions

Sensor	Performance description	Sensor	Performance description
S1	Ammonia and amines	S8	Volatile organic compounds
S2	Hydrogen sulfide and sulfides	S9	Hydroxides, gasoline, and kerosene
S3	Hydrogen	S10	Alkanes and combustible gases
S4	Alcohols and organic solvents	S11	Aromatic compounds
S5	Cooking odors and volatile organic compounds	S12	Sulfides
S6	Methane, biogas, and hydrocarbons	S13	Sterols and triterpenoids
S7	Combustible gases	S14	Lactones and pyrazines

Table S2. Basic physicochemical properties of kiwifruit wine

Group	pH	Soluble solids	Total Acidity (g/L)	Alcohol Content (%, v/v)	Reducing Sugars (g/L)
CK	3.68 ± 0.06^{c}	7.03 ± 0.10^{a}	12.37 ± 0.23^{c}	10.70 ± 0.12^{a}	2.02 ± 0.04^{b}
B1	3.83 ± 0.01^{b}	6.89 ± 0.04^{abc}	12.47 ± 0.16^{bc}	10.67 ± 0.10^{a}	2.16 ± 0.03^{a}
B2	3.89 ± 0.03^{a}	6.81 ± 0.12^{bc}	12.72 ± 0.15^{ab}	10.81 ± 0.11^{a}	2.03 ± 0.09^{b}
B3	3.84 ± 0.01^{ab}	6.90 ± 0.10^{ab}	12.88 ± 0.04^{a}	10.71 ± 0.06^{a}	1.99 ± 0.05^{b}
B4	3.89 ± 0.02^{a}	6.71 ± 0.09^{c}	12.81 ± 0.11^{a}	10.75 ± 0.07^{a}	1.98 ± 0.06^{b}

Table S3. Odor activity values of key aroma-active compounds identified in kiwifruit wine

No.	Compound	Odor threshold (mg/L)	Odor characteristic	OAV				
				CK	B1	B2	B3	B4
1	Isoamyl acetate	0.017	Banana, sweet, fruity	163.63±29.47[c]	217.3±12.21[b]	267.14±37.2[ab]	295.78±35.29[a]	129.32±22.52[c]
2	Ethyl hexanoate	0.001	Fruity, apple, pineapple	27.69±4.10[c]	47.55±4.72[b]	52.92±7.76[ab]	61.33±10.56[a]	29.28±7.01[c]
3	Hexyl acetate	0.002	Fruity, apple, green	1.78±1.20[a]	2.28±0.56[a]	1.16±0.37[a]	1.84±0.73[a]	1.02±0.33[a]
4	Ethyl heptanoate	0.002	Fruity, pineapple	2.31±1.32[a]	2.24±0.21[a]	0.84±0.61[a]	1.68±0.82[a]	1.29±0.46[a]
5	Hex-2-enoic acid ethyl ester	0.015	Green, fruity	2.11±0.60[a]	1.59±0.39[ab]	1.84±0.46[ab]	1.08±0.19[b]	2.02±0.25[a]
6	Ethyl octanoate	0.002	Fruity, floral, sweet	126.36±12.65[b]	155±21.49[b]	285.16±38.02[a]	289.05±51.04[a]	292.29±42.52[a]
7	Propyl n-Octanoate	0.012	Fruity	2.46±0.36[a]	1.47±0.47[b]	—	—	0.76±0.29[c]
8	Ethyl nonanoate	0.001	Fatty, floral, fruity	1.68±0.77[b]	3.87±0.43[a]	2.2±0.62[b]	1.44±0.42[b]	1.23±0.49[b]
9	Ethyl decanoate	0.2	Fruity, grape, oily	93.43±9.88[b]	99.61±5.78[b]	107.15±10.39[b]	129.65±16.26[a]	108.36±11.92[b]
10	Isoamyl octanoate	0.002	Fruity	6.80±1.32[a]	10.16±4.68[a]	11.66±2.24[a]	12.83±0.51[a]	10.97±4.58[a]
11	Ethyl 9-decenoate	0.001	Fruity, pear	12.93±0.77[a]	10.77±2.43[ab]	9.85±1.92[abc]	8.61±1.28[bc]	6.69±2.15[c]
12	Phenethyl acetate	0.25	Rose, floral, fruity	33.02±4.58[a]	24.01±2.51[b]	25.49±4.63[ab]	33.35±5.08[a]	27.93±3.66[ab]
13	Isoamyl alcohol	0.3	Fusel, malty	263.08±16.36[a]	263.79±15.65[a]	237.38±16.00[a]	265.25±17.33[a]	194.04±8.63[b]
14	1-Hexanol	0.5	Green, resinous	54.70±5.19[b]	43.75±5.68[b]	53.49±7.55[b]	69.87±9.82[a]	46.05±10.81[b]
15	1-Heptanol	0.3	Earthy, green, citrus	18.61±4.87[ab]	14.94±3.11[b]	21.79±6.33[ab]	23.76±3.05[a]	17.55±3.41[ab]
16	2-Ethylhexanol	0.015	Citrus, floral	3.93±1.53[a]	3.75±0.61[a]	3.60±0.48[a]	5.49±0.19[a]	3.87±1.81[a]
17	1-Octanol	0.13	Artificial, waxy, green	20.06±3.84[ab]	15.92±5.03[b]	28.38±4.94[a]	27.23±0.59[a]	22.52±5.6[ab]

Table S3 (Continued)

No.	Compound	Odor threshold (mg/L)	Odor characteristic	OAV				
				CK	B1	B2	B3	B4
18	Trans-2-octen-1-ol	0.01	Green, fatty	5.88±1.01[ab]	4.68±0.95[b]	4.88±1.77[b]	7.61±1.30[a]	5.73±1.19[ab]
19	1-Nonanol	0.05	Fatty, floral, green	4.81±0.77[ab]	3.79±1.21[b]	4.21±1.04[ab]	5.61±0.71[a]	5.17±0.71[ab]
20	Decyl alcohol	0.01	Fatty, floral	9.61±0.56[a]	9.34±0.88[a]	4.92±0.82[b]	—	8.45±2.01[a]
21	1-Octen-3-ol	0.001	Mushroom, earthy	—	8.54±2.38[ab]	13.37±9.69[a]	15.19±1.69[a]	10.84±4.37[a]
22	Linalool	0.015	Floral, citrus, lavender	—	2.49±1.03[b]	2.22±1.11[b]	—	4.98±0.65[a]
23	cis-3-Hexen-1-ol	0.07	Green, leafy	1.74±0.13[b]	2.09±0.78[b]	1.93±0.47[b]	5.94±0.14[a]	2.71±1.26[b]
24	2-Octanone	0.05	Earthy, mushroom, blue cheese	101.06±12.44[c]	113.21±5.23[bc]	137.84±21.43[ab]	156.91±16.81[a]	88.07±25.75[c]
25	2-Nonanone	0.05	Blue cheese, fruity	3.14±1.05[a]	1.15±0.46[b]	1.92±0.28[ab]	3.06±0.64[a]	1.93±0.83[ab]
26	2-Undecanone	0.05	Floral, waxy, citrus	4.1±1.42[ab]	4.37±0.41[a]	3.97±1.53[ab]	2.16±0.37[bc]	1.67±1.12[c]
27	Menthone	0.05	Minty, cooling	3.66±0.75[a]	—	—	4.01±0.38[a]	3.3±1.11[a]
28	3-Octanone	0.05	Mushroom, earthy	—	—	3.16±0.29[a]	4.00±0.66[a]	1.84±0.77[b]
29	β-Damascenone	0.002	Apple, rose, plum	—	1.34±0.57[a]	1.01±0.53[a]	—	—
30	Octanoic acid	0.5	Fatty, cheesy	98.64±14.1[a]	80.65±4.53[bc]	67.47±6.87[c]	83.73±5.99[b]	68.07±4.47[c]
31	4-Vinylphenol	0.01	Herbaceous, spicy	6.32±1.53[a]	5.24±0.43[a]	3.84±0.56[a]	5.01±0.96[a]	6.48±2.86[a]

Table S4. Relative abundance of significantly differential metabolites

No.	Compound	VIP value	Relative abundance(×10^6)	
			CK	B3
1	Quinic acid	33.42	27102.2±596.2	22243.8±807.1**
2	Succinic acid	25.70	5836.1±101.8	9523.1±329.2**
3	Octadecanamide	24.12	2712.3±264.0	14178.8±1352.8**
4	Leucine	8.79	1029.9±22.8	1477.0±211.8
5	Uridine	6.15	942.1±20.4	1185.1±141.8
6	2-Aminooctadecane-1,3,4-triol	6.09	3279.9±58.9	3830.7±358.3
7	Phenylalanine	5.84	1197.8±57.6	1550.1±186.6
8	L-Leucyl-L-proline	3.73	939.0±45.3	118.5±67.8*
9	10-Hydroxydecanoic acid	3.61	270.3±18.4	327.1±47.3
10	gamma-Glu-leu	3.45	356.3±12.1	404.5±38.0
11	Caffeic acid	3.35	2837.4±231.1	3110.5±242.0
12	8-Hydroxy-9,10-epoxystearic acid	2.82	203.9±18.4	160.7±8.7*
13	Leu-Gly-Gly	2.77	138.7±3.8	163.0±15.3
14	Fructose (Generic Ketohexose)	2.77	11.3±4.6	48.5±16.5
15	gamma-Linolenic acid	2.66	135.9±12.6	103.7±6.5*
16	Rosmarinic acid	2.63	557.6±27.7	637.4±37.1*
17	Myristic acid	2.42	104.5±11.4	69.0±11.6*
18	Galactaric acid	2.38	89.4±3.9	124.3±14.2*
19	Neochlorogenic acid	2.32	186.6±25.2	233.9±18.9
20	delta10-12-PhytoF	2.25	130.0±7.3	159.4±13.2*
21	Caffeic acid hexoside	2.19	74.12±2.1	159.4±13.2*
22	beta-Gentiobiose	2.12	61.8±5.9	81.6±3.4*
23	Guanine	2.01	94.7±3.2	107.5±9.2
24	9-Hydroperoxyoctadeca-10,12-dienoic acid	1.99	127.9±7.0	113.7±2.2
25	N-Fructosyl phenylalanine	1.96	109.7±10.9	19.2±9.3*
26	Tryptophol	1.89	158.8±14.6	193.1±19.0
27	3-p-Coumaroylquinic acid	1.75	43.7±0.7	34.4±2.3*
28	Acacetin-7-O-rutinoside	1.69	11.3±3.0	20.4±1.7*
29	2,3-Dihydroxy-3-methylbutanoic acid	1.69	39.4±11.2	66.7±7.9*

Table S4 (Continued)

No.	Compound	VIP value	Relative abundance(×10^6)	
			CK	B3
30	Gulonic acid	1.69	106.0±4.2	124.4±12.6
31	2-Palmitoylglycerol	1.65	31.7±4.6	40.7±3.4
32	L-Histidinol	1.62	72.1±5.8	57.4±2.3*
33	12-Hydroxydodecanoic acid	1.56	79.6±3.0	71.7±0.2*
34	Coumarin	1.51	38.4±4.1	26.2±3.1*
35	3-Methyladipic acid	1.46	46.0±6.1	67.6±3.5*
36	FA 18:3;3O	1.43	46.0±4.4	57.0±2.8*
37	Phenylpyruvic acid	1.38	26.1±0.3	20.0±1.8*
38	3'-O-Methylguanosine	1.38	23.7±1.5	28.9±1.8*
39	D-Glucosamine	1.29	60.8±4.9	90.6±8.2*
40	Glycyl-L-proline	1.16	10.8±0.7	19.7±1.7**
41	Maltol	1.05	25.4±0.1	32.5±2.3*
42	Pimelic acid	1.03	10.3±1.3	7.8±1.0
43	Cyclo-prolylglycine	1.01	15.4±0.9	19.7±0.4**

Note: VIP values were derived from the OPLS-DA model; Group comparisons were performed using Student's *t*-test. * $p < 0.05$, ** $p < 0.01$.
